# Supplementary material for: The Systems Biology Research Tool: evolvable open-source software
Source: BMC Syst Biol. 2008 Jun 29;2:55. doi: 10.1186/1752-0509-2-55 (PMC2446383; doi:10.1186/1752-0509-2-55)
Supplement: Additional file 1 — SBRT Archive. An archive of the current version of the Systems Biology Research Tool. [file 1752-0509-2-55-S1.zip › sbrt-1.4.0/doc/developers_guide/api/sbrt/kernel/fba/proc/SvCdHarFluxVectorProcess.html]

SvCdHarFluxVectorProcess


|  |  |  |
| --- | --- | --- |
| **Overview** \| **Package** \| **Class** \| **Use** \| **Tree** \| **Deprecated** \| **Index** \| **Help** | |  |
| **PREV CLASS**   **NEXT CLASS** | **FRAMES**    **NO FRAMES**     **All Classes** |  |
| SUMMARY: NESTED \| FIELD \| CONSTR \| METHOD | DETAIL: FIELD \| CONSTR \| METHOD |  |


---


## sbrt.kernel.fba.proc Class SvCdHarFluxVectorProcess

```
java.lang.Object
  sbrt.kernel.fba.proc.FbaProcess<R>
      sbrt.kernel.fba.proc.CvFbaOptProcess<CdHarFluxVectorComputer.Results>
          sbrt.kernel.fba.proc.SvCdHarFluxVectorProcess
```

**All Implemented Interfaces:**: KernelProcess<CdHarFluxVectorComputer.Results>, MonitoredProcess<CdHarFluxVectorComputer.Results>

---

``` public final class SvCdHarFluxVectorProcess extends CvFbaOptProcess<CdHarFluxVectorComputer.Results> implements MonitoredProcess<CdHarFluxVectorComputer.Results> ```

This class is used to perform space variation coordinate direction
hit-and-run flux vector experiments.

**Author:**
:   This class was written and documented by Jeremiah Wright while in
    the Wagner lab.

---

**Constructor Summary**

`SvCdHarFluxVectorProcess(CdHarFluxVectorComputer computer)`
  
          Constructs a new constraint variation coordinate direction hit-and-run
flux vector experiment.


| **Method Summary** | |
| --- | --- |
| `Fluxome` | `getFluxome()`             Returns the fluxome for which flux vectors are to be generated. |
| `ProgressMonitor` | `getProgress()`             Returns a progress monitor for this experiment. |
| `void` | `run(ResultsManager<CdHarFluxVectorComputer.Results> resultsManager)`             Runs this process and adds the generated results to the provided results manager via its `ResultsManager.addData(Object)` method. |
| `void` | `setConstraintSource(java.lang.Iterable<FluxConstraints> constraintSource)`             Sets the constraint variation input source. |
| `void` | `setFluxVectorSource(java.lang.Iterable<java.util.Map<java.lang.String,java.lang.Double>> fluxVectorSource)`             Sets the source of flux vectors to serve as initial points. |
| `void` | `setIterations(int iterations)`             Sets the total number of flux vectors to be generated. |

**Methods inherited from class sbrt.kernel.fba.proc.CvFbaOptProcess**

`restoreConstraints, setConstraints`

**Methods inherited from class sbrt.kernel.fba.proc.FbaProcess**

`toString`

**Methods inherited from class java.lang.Object**

`clone, equals, finalize, getClass, hashCode, notify, notifyAll, wait, wait, wait`

**Constructor Detail**

### SvCdHarFluxVectorProcess

```
public SvCdHarFluxVectorProcess(CdHarFluxVectorComputer computer)
```

:   Constructs a new constraint variation coordinate direction hit-and-run
    flux vector experiment.

    **Parameters:**: `computer` - the computer with which all flux vectors will be generated.


**Method Detail**

### setIterations

```
public void setIterations(int iterations)
```

:   Sets the total number of flux vectors to be generated.

    :   **Parameters:**: `iterations` - the total number of flux vector to be generated.

---


### getFluxome

```
public Fluxome getFluxome()
```

:   Returns the fluxome for which flux vectors are to be generated.

    :   **Specified by:**: `getFluxome` in class `FbaProcess<CdHarFluxVectorComputer.Results>`
    :   **Returns:**: the fluxome for which flux vectors are to be generated.

---


### getProgress

```
public ProgressMonitor getProgress()
```

:   Returns a progress monitor for this experiment.

    :   **Specified by:**: `getProgress` in interface `MonitoredProcess<CdHarFluxVectorComputer.Results>`
    :   **Returns:**: a progress monitor for this experiment.

---


### setConstraintSource

```
public void setConstraintSource(java.lang.Iterable<FluxConstraints> constraintSource)
```

:   Sets the constraint variation input source.

    :   **Parameters:**: `constraintSource` - a source of constraint sets whose effect upon application is to be computed.

---


### setFluxVectorSource

```
public void setFluxVectorSource(java.lang.Iterable<java.util.Map<java.lang.String,java.lang.Double>> fluxVectorSource)
```

:   Sets the source of flux vectors to serve as initial points.

    :   **Parameters:**: `fluxVectorSource` - a source of initial points.

---


### run

```
public void run(ResultsManager<CdHarFluxVectorComputer.Results> resultsManager)
```

:   Runs this process and adds the generated results to the provided results
    manager via its `ResultsManager.addData(Object)` method.

    First, a set of flux constraints is retrieved from the constraint source
    and applied. Then an initial point is retrieved from the flux vector
    source and set. The random flux vectors are then computed and added to
    the provided results manager. Finally, the flux constraints are restored to
    their original values. This process is repeated until the ends of the
    constraint and flux vector sources are reached.

    :   **Specified by:**: `run` in interface `KernelProcess<CdHarFluxVectorComputer.Results>`
    :   **Parameters:**: `resultsManager` - the results manager to which the results will be added.


---


|  |  |  |
| --- | --- | --- |
| **Overview** \| **Package** \| **Class** \| **Use** \| **Tree** \| **Deprecated** \| **Index** \| **Help** | |  |
| **PREV CLASS**   **NEXT CLASS** | **FRAMES**    **NO FRAMES**     **All Classes** |  |
| SUMMARY: NESTED \| FIELD \| CONSTR \| METHOD | DETAIL: FIELD \| CONSTR \| METHOD |  |


---
